# Sex-specific outcomes in cancer therapy: the central role of hormones

**DOI:** 10.3389/fmedt.2024.1320690

**Published:** 2024-02-01

**Authors:** Parisa Bakhshi, Jim Q. Ho, Steven Zanganeh

**Affiliations:** ^1^ Research and Development, MetasFree Biopharmaceutical Company, Mansfield, MA, United States; ^2^ Department of Internal Medicine, Yale School of Medicine, New Haven, CT, United States

**Keywords:** sex hormone, sex-specific oncology, cancer therapy, personalized medicine, sex-specific medicine

## Abstract

Sex hormones play a pivotal role in modulating various physiological processes, with emerging evidence underscoring their influence on cancer progression and treatment outcomes. This review delves into the intricate relationship between sex hormones and cancer, elucidating the underlying biological mechanisms and their clinical implications. We explore the multifaceted roles of estrogen, androgens, and progesterone, highlighting their respective influence on specific cancers such as breast, ovarian, endometrial, and prostate. Special attention is given to estrogen receptor-positive (ER+) and estrogen receptor-negative (ER−) tumors, androgen receptor signaling, and the dual role of progesterone in both promoting and inhibiting cancer progression. Clinical observations reveal varied treatment responses contingent upon hormonal levels, with certain therapies like tamoxifen, aromatase inhibitors, and anti-androgens demonstrating notable success. However, disparities in treatment outcomes between males and females in hormone-sensitive cancers necessitate further exploration. Therapeutically, the utilization of hormone replacement therapy (HRT) during cancer treatments presents both potential risks and benefits. The promise of personalized therapies, tailored to an individual’s hormonal profile, offers a novel approach to optimizing therapeutic outcomes. Concurrently, the burgeoning exploration of new drugs and interventions targeting hormonal pathways heralds a future of more effective and precise treatments for hormone-sensitive cancers. This review underscores the pressing need for a deeper understanding of sex hormones in cancer therapy and the ensuing implications for future therapeutic innovations.

## 1 Introduction

Sex hormones, such as androgens and estrogens, are pivotal in dictating the physiological and morphological differences between males and females (1). These hormones extend their influence beyond reproduction, significantly modulating a myriad of pathological processes, including cancer (2). Over time, the conventional understanding of steroid hormones as transcription factors that predominantly regulate reproductive organs has changed (3). Sex steroid receptors, including estrogen (ER), progesterone (PR), and androgen (AR) receptors, were initially perceived as transcription factors governing physiological and pathological responses in reproductive organs (4). Upon hormone binding, these receptors were thought to translocate to the nucleus, recognizing hormone-responsive elements (HREs) and regulating gene transcription (5). However, recent studies have revealed different roles for steroid receptors that includes both non-genomic and genomic pathways (6). Beyond their classical transcriptional functions, steroid receptors can rapidly activate transduction pathways, such as PI3K/AKT or MAPKs, influencing various physiological and pathological processes in diverse anatomical sites (7). This paradigm shift in understanding suggests that steroid receptors play a broader role in regulating key genes, impacting organ development, function, and, notably, contributing to the development and progression of cancers (8, 9). While classical hormone-related cancers like breast, prostate, and ovary have been extensively studied, a growing body of research investigates the influence of sex steroid receptors in various cancers (10). Researchers are exploring the complex relationships between steroid hormones and their receptors and the prevalence of cancer in both men and women (6).

**Figure 1:**
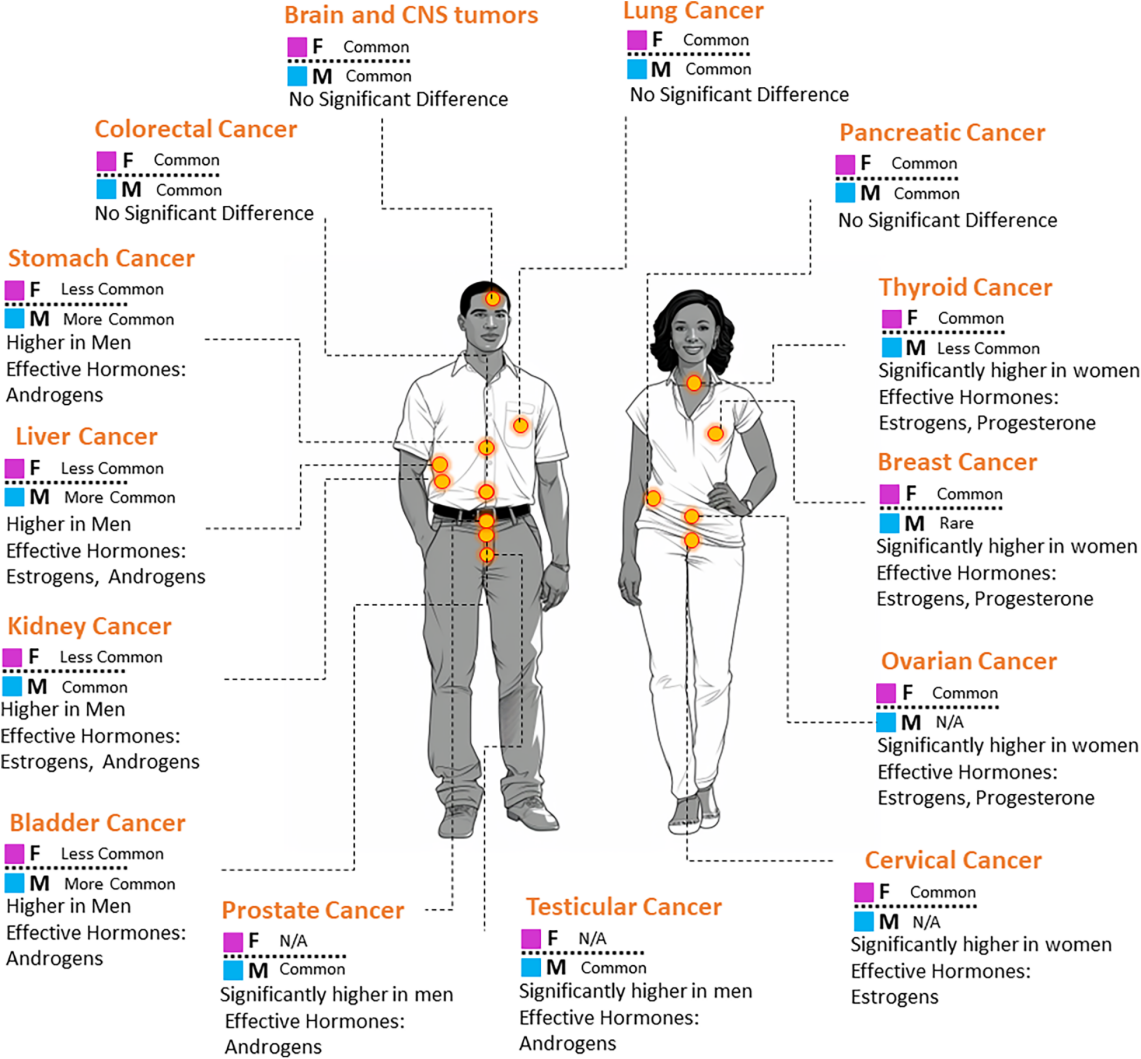
A visual representation comparing the prevalence of select cancer types in males and females. The associated key hormones with potential influence on each cancer are also indicated.

Looking further into the extensive literature surrounding sex hormones and cancer, reveals that these biological factors have profound influence beyond their well-established roles in sexual development (2, 11, 12). Sex hormones emerging as key players in the complex landscape of cancer. As illustrated in Figure 1, the incidence of specific cancers varies between males and females. Such disparities are often attributed to the influence of sex hormones (13). The nuanced interplay between sex hormones and cellular pathways in different tissues can either augment or suppress cancer's onset, progression, and response to therapies (14). Consequently, this interaction often manifests as marked differences in cancer outcomes across sexes.

Androgens, predominantly testosterone, produced mainly by the testes and in smaller quantities by the adrenal glands, drive the development of male reproductive tissues and the manifestation of secondary sexual characteristics (15). Notably, prostate cancer exhibits a profound association with androgen levels, rendering androgen deprivation therapy a therapeutic mainstay for affected individuals (16). Elevated androgen levels also harbor implications for liver cancer (17, 18) and have hinted at roles in bladder, kidney, lung, and breast malignancies (19, 20).

Conversely, estrogens, chiefly estradiol, produced in the ovaries, with contributions from the adrenal glands and adipose tissues, are essential for female reproductive organ growth, menstrual cycle regulation, and secondary sexual characteristic manifestation (21). In oncology, heightened estrogen levels are unequivocally linked to breast tumor proliferation (22). Additionally, the coordinated action of estrogens and their specific receptors plays a critical role in the pathogenesis of ovarian and endometrial cancers, highlighting their multifaceted influence on female-centric malignancies (23–25).

However, the scope of these hormones transcends sex-specific cancers. Intriguingly, estrogen-related pathways have been identified as potential targets for improving immunotherapy responses in melanoma, a skin cancer unconfined to sex-specific occurrence (26). Moreover, novel insights suggest that estrogen signaling, particularly in myeloid cells, fosters immune suppression in melanoma, and targeting ERα could further enhance immunotherapeutic outcomes (23). Concurrently, interventions combining radiation therapy and Fulvestrant, an estrogen receptor antagonist, have shown to bolster immunotherapy responses in ER + breast cancer (27). Such revelations accentuate the expanding horizon of hormonal influences in cancer therapy (28).

The literature also highlights the importance of the immune microenvironment and its interactions with estrogens and their receptors in shaping therapeutic outcomes in breast cancer (25, 29). Hormone receptor antagonism has been shown to amplify the expression of immunotherapeutic targets on breast tumors, suggesting a potential synergistic approach for treatment (30). Furthermore, emerging evidence underscores the role of β-estradiol in non-small cell lung cancer’s tumorigenesis, prognosis, and therapeutic responses (31, 32). Sex differences in immunotherapy responses are evident, necessitating exploratory endeavors for novel therapeutic combinations (33, 34). The synthesis, secretion, and equilibrium of these hormones can be swayed by a constellation of factors including age, metabolic status, and environmental exposures (35, 36). As we navigate the intricate nexus of sex hormones and their omnipresence in the oncological landscape, it becomes palpably clear that a comprehensive understanding of these dynamics is not just requisite but instrumental in paving the way for tailored, sex-specific therapeutic paradigms, heralding a new epoch in cancer care (37–39). Recognizing the profound influence of sex hormones on immune modulation, researchers are harnessing immunotherapy's potential to tailor treatments based on hormonal profiles. Such strategies have shown promise, particularly when targeting hormone-responsive cancers, underscoring the significance of understanding hormone-immune system interplay (40–47).

## 2 Biological mechanisms

### 2.1 Estrogen and its influence on cancer progression

Estrogen, often used as a collective term for several related hormones, plays crucial roles in numerous physiological functions, particularly within the reproductive and cardiovascular systems (48). Beyond these functions, estrogen's interactions at cellular levels have implications in oncology (49). Estrogen exerts its effects primarily through its binding with specific intracellular receptors, specifically estrogen receptor alpha (ER*α*) and estrogen receptor beta (ERβ) (50). Such interactions initiate a cascade of gene transcription events that in turn regulate protein synthesis and dictate cellular actions vital for tissue morphogenesis, cellular proliferation, differentiation, and apoptosis; processes intrinsically linked with oncogenesis (2, 25).

Estrogen acts via both non-genomic and genomic signaling mechanisms (51). The actions can be mediated either by processes that do not involve direct binding to DNA (non-genomic effects) or by direct binding of estrogen receptor complexes to particular regions in gene promoters (genomic effects). Direct genomic signaling is considered the classical mechanism of estrogen signaling, and non-genomic signaling is considered the non-classic mechanism (52).

In genomic signaling, estrogen binds to nuclear receptors (ERα and ERβ), inducing conformational changes that lead to dimerization and translocation to the nucleus. There, the receptors bind to estrogen response elements (EREs) on DNA, regulating gene expression directly (53). However, not all estrogen-regulated genes have EREs, and approximately 35% of estrogen-responsive genes lack them. When it comes to indirect genomic signaling, estrogen receptors interact with transcription factors like the activator protein (AP)-1, nuclear factor-κB (NF-κB) and stimulating protein-1 (Sp-1), stimulating gene expression without direct DNA binding. This mechanism involves protein-protein interactions and influences target genes such as LDL receptor, progesterone receptor, and endothelial nitric oxide synthase (54).

Non-genomic signaling, on the other hand, operates outside the nucleus and involves rapid responses (51). The GPER1 receptor and variants of ERα and ERβ are associated with this pathway. Estrogen activates signal-transduction cascades, including PLC/PKCs, Ras/Raf/MAPK, PI3K/Akt, and cAMP/PKA pathways. These cascades result in the phosphorylation of transcription factors, affecting gene expression indirectly. The membrane receptors ERα and ERβ may interact with scaffold proteins and membrane receptors, activating signaling cascades and influencing transcriptional regulation (50, 55).

The crosstalk between genomic and non-genomic pathways involves complex interactions between nuclear receptors, membrane receptors, and various transcription factors. Two proposed mechanisms include the dimerization of nuclear estrogen receptor complexes with phosphorylated transcription factors and the activation of protein kinase cascades at the plasma membrane, leading to enhanced transcriptional activity (56). Furthermore, estrogen receptors can be activated independently of ligands, triggered by phosphorylation events involving protein kinases, inflammatory cytokines, cell adhesion molecules, and growth factors. In summary, estrogen signaling is a multifaceted process involving both genomic and non-genomic pathways, with intricate crosstalk mechanisms contributing to the regulation of gene expression in various cellular contexts (50).

Breast cancer serves as a prime example of estrogen's significant influence on oncogenesis. Estrogen-responsive breast cancer cells utilize the hormone's interaction with ER*α* to promote cell cycle progression and inhibit apoptosis (57). Furthermore, estrogen boosts the expression of certain growth factors, including insulin-like growth factor (IGF) and vascular endothelial growth factor (VEGF), enhancing tumor growth and angiogenesis (58, 59). About 75% of breast cancer tumors express ERα (60). Treatments for patients with these ERα-positive tumors typically include selective ER modulators (e.g., Tamoxifen) (61), selective ER degraders (e.g., Fulvestrant) (62), or aromatase inhibitors (63). Even though these treatments can effectively restrict ER-positive breast cancer cell growth, prolonged ERα inhibition may stimulate immunosuppressive activities in these cells, triggering several immune checkpoint processes (64, 65). Hormonal therapies, especially those targeting ER+, are used in treating tumors that predominantly rely on estrogenic signaling. On the contrary, ER- tumors, lacking these receptors, tend to be more aggressive and require tailored therapeutic strategies (66–68).

Elevated estrogen levels correlate with an increased risk of ovarian cancer. The oncogenic properties of estradiol, a form of estrogen, are particularly concerning when considering the adverse effects of estrogen-only treatments during menopause (69). Several studies have also indicated that estrogen may promote the proliferation and migration of ovarian cancer cells (70). The deeper mechanistic relationship between elevated estrogen levels and increased ovarian cancer risk is still under investigation (53, 54). Estrogen’s influence is also evident in endometrial cancer. Prolonged exposure to estrogen without the balancing effect of progesterone can heighten the risk of this type of cancer (71, 72). In the context of prostate cancer, prevailing treatments can sometimes result in castration-resistant forms of the disease. Targeting specific ER subtypes (α or β) offers a promising avenue for mitigating the growth and spread of prostate cancer cells. Notably, ERβ has been identified as a potential tumor suppressor, suggesting its activation could be leveraged in therapeutic strategies against prostate cancer (73).

### 2.2 Androgens and cancer growth

Androgens, primarily testosterone and its derivatives, play foundational roles in male physiology, governing secondary sexual features, reproductive capacities, and bone health (74). Testosterone, produced by Leydig cells in response to luteinizing hormone, activates androgen receptor (AR) signaling in Sertoli cells (SCs). The AR signaling pathway can be categorized into classical and non-classical pathways (75).

In the classical pathway, androgen binding to cytoplasmic AR leads to nuclear translocation, dimerization, and binding to androgen response elements (AREs) to regulate gene transcription. This process is relatively slow, requiring 30–45 min for transcriptional changes (75).

The non-classical pathway involves membrane-bound AR, rapid activation within 1 min, and subsequent activation of kinases such as Src, ERK, and Akt. These pathways play roles in regulating gene expression, spermatogenesis, and cellular processes (76). Three distinct non-classical signaling pathways associated with testosterone action have been identified in the testis. The first and main pathway involves the binding of testosterone to membrane-bound AR, interacts with the SH3 domain of the SRC proto-oncogene (Src), and triggers a cascade involving EGFR, Ras kinase, MAPK cascades (Ras-Raf-MEK-ERK), and downstream transcription factors like cAMP-response element-binding protein (CREB) (77). Another non-classical pathway involves the PI3K/Akt pathway, activated by the PI3K subunit p85α. An additional non-classical pathway specific to immature Sertoli cells induces depolarization within the K+ ATP channels, mediated by G protein-induced activation of phospholipase C, resulting in a quick influx of Ca2+ and subsequent activation of signaling molecules (75, 78, 79).

Beyond these primary functions, androgens have a pronounced footprint in oncology, especially in prostate and breast cancers, and are further implicated in other malignancies (80, 81). The prostate gland is intricately sensitive to androgens, with cellular interactions predominantly mediated through the AR. When androgens bind to the AR, it triggers the activation of the receptor, facilitating its transportation into the cell nucleus (82). Consequently, this process plays a crucial role in modulating gene transcription, which is vital for cellular growth and survival. This physiologically essential pathway can, however, shift to pathological roles, especially in early stages of prostate cancer that exhibit androgen-driven growth. Such observations underlie the therapeutic approach of androgen deprivation therapy (ADT) for prostate cancer (83).

Beyond the prostate, the story of androgens grows more complex. Dihydrotestosterone (DHT), a powerful derivative of testosterone, has been shown to stimulate certain types of breast cancer cells, suggesting an androgenic influence in breast tumor biology (80, 81). Post-menopausal increases in systemic androgen levels have also been linked to heightened risks of endometrial cancer, possibly via indirect pathways that bolster estrogen synthesis (84, 85). The AR has been meticulously studied in cancers that display hormone sensitivity, like the prostate and breast cancers mentioned. Cancers that are AR-positive may depend on androgens for growth and progression. Research hints at the significance of AR signaling even in some hormone-independent cancers. For instance, male prevalence in liver and stomach cancers exceeds that of females, suggesting potential hormonal influence (86). This observation extends to other malignancies like bladder, kidney, pancreas, liver, endometrial, specific lymphomas, and salivary gland cancers, in which AR signaling might display varied effects (19, 87).

With advancing research uncovering the diverse roles of AR, there's potential for developing treatments that target either AR directly or its downstream signaling pathways. This perspective offers promising avenues for cancer therapy (88). Additionally, recent clinical insights suggest that ADT might enhance the effectiveness of some immunotherapies, including immune checkpoint inhibitors (89).

### 2.3 Progesterone and cancer interactions

A cornerstone for female reproductive processes, progesterone also occupies a pivotal position in cancer biology. Progesterone acts through both genomic (classical) and nongenomic (non-classical) signaling pathways, affecting different tissues and potentially having an impact on the development of cancer (90).

Classical progesterone receptors (PRs), including PR-A and PR-B isoforms, operate as nuclear receptors, initiating transcriptional changes upon ligand binding. These receptors undergo conformational changes, dimerize, translocate to the nucleus, and bind to progesterone response elements (PREs) to regulate target gene transcription (91). Various PR isoforms, such as PR-A, PR-B, PR-C, PR-M, PR-S, and PR-T, exhibit tissue-selective signaling with distinct roles in organs like the mammary gland and uterus. In classical signaling, progesterone plays a crucial role in mammary gland proliferation, impacting pathways like RANKL, CCND1 (Cyclin D1), and WNT-1. PR-B signaling is predominant in normal mammary gland function, while PR-A is crucial for uterine and ovarian functions. Prolonged exposure to progesterone, as observed during the luteal phase, may lead to dysregulated pathways and contribute to breast cancer (92, 93).

The non-classical progesterone signaling, highlighting rapid progestin-activated pathways that involve membrane-associated actions on EGFR, c-Src, and MAPK (94). Membrane progesterone receptors (mPRs), a class of proteins that resemble G protein-coupled receptors (GPCRs) in structure, mediate non-classical signaling. The five mPRs (mPRα, mPRβ, mPRɣ, mPRε, and mPRδ) activate MAPKs, ERK1/2, and intracellular Ca2+ influx. Additionally, membrane-associated progesterone receptors, PGRMC1 and PGRMC2, contribute to non-classical signaling, influencing cholesterol synthesis, cytochrome P450 (CYP) enzymes, and intracellular heme transport (93, 95).

Progesterone roles in oncology are multifaceted, often marked by a duality wherein it can either support or counteract cancer progression, contingent upon its complex signaling interplay (96). In the realm of breast cancer, progesterone occasionally stimulates cellular growth, particularly when its receptors engage with certain growth factors (97). However, in a contrasting light, there are scenarios in which progesterone acts against breast cancer by predominantly steering breast cell differentiation (39). Interactions with other hormonal pathways, especially estrogen, further complicate progesterone’s role. Often, it acts to temper estrogen-driven cell proliferation in tissues, especially in the endometrium. Any disturbances in this intricate balance, be it due to a deficit of progesterone or an excess of estrogen, can amplify risks associated with endometrial cancer (98).

A pivotal aspect of progesterone's interaction with cancer involves its interplay with other signaling pathways. Notably, its communication with growth factors like insulin-like growth factor (IGF) can modify cellular hormonal responses, a comprehension of which can inform targeted therapeutic approaches (38). When discussing colon cancer, combined actions of estradiol and progesterone appear to be instrumental. Their collective activity is posited to inhibit tumor proliferation and induce apoptosis, perhaps through the activation of ERβ (99). Research into progesterone's effects on prostate cancer (100), breast cancer (97), and ovarian cancer (101) paints a complex picture with findings that are, at times, contradictory or multifarious in interpretation.

As depicted in Table 1, a summary of the sex hormone signaling pathways, focusing on estrogen, androgen, and progesterone was provided.

**Table 1:** Summary of sex hormone signaling pathways.

Hormone	Signaling pathways	Genomic pathway	Non-genomic pathway	Intracellular molecules	Target tissues	Main effects	References
Estrogen	Estrogen receptor (ER)	ERα/ERβ, gene transcription	Rapid activation of signaling	PI3K/Akt, MAPK/ERK, SRC	Uterus, mammary glands, bones	Regulation of menstrual cycle, bone health, breast development, cardiovascular protection	(50, 52, 54)
Testosterone	Androgen receptor (AR)	AR, gene transcription	Rapid activation of signaling	PI3K/Akt, MAPK/ERK, JAK/STAT, SRC	Testes, muscle, bone	Spermatogenesis, muscle development, bone health, libido, secondary sexual characteristics	(75)
Progesterone	Progesterone receptor (PR)	PR, gene transcription	Rapid activation of signaling	PI3K/Akt, MAPK/ERK, NF-*κ*B, SRC	Uterus, mammary glands, brain	Regulation of menstrual cycle, pregnancy support, breast development, neurotransmission	(93, 95)

The presented table provides summary of sex hormone signaling pathways in cancer therapy. This table presents an overview of the key aspects of sex hormone signaling, including main effects, target tissues, involved intracellular molecules, and the distinct genomic and non-genomic pathways associated with estrogen, androgen, and progesterone. It also delineates the specific signaling pathways activated by these hormones, underlining their crucial roles in cancer development and treatment outcomes.

## 3 Clinical outcomes and observations

Cancer treatment outcomes can vary greatly. Although numerous elements play a role, recent research has highlighted the crucial role of sex hormone levels in determining treatment efficacy (37). This intersection of hormones and therapeutic responses has rapidly emerged as a key area of focus. Table 2 offers a detailed summary of various sex hormones and their links with particular cancers.

**Table 2:** Overview of sex hormones and their associations with various cancers.

Sex Hormones	Associated cancers	Role in normal physiology	Mechanism of action	Receptor status	Treatment implication	Potential prevention measures	Common age of onset (years)	References
Estrogens (E2)	Breast	Regulate female reproductive system	Stimulates ER+ breast cancer cell proliferation	ER+ ∼70% of cases	Tamoxifen, aromatase inhibitors	Regular screening, reduce HRT	50–70	(102, 103)
	Endometrial	Regulate female reproductive system	Prolonged exposure can lead to hyperplasia	ER+Common in type	Progestins, Hysterectomy	Reduce prolonged unopposed E2	55–65	(104, 105)
Androgens (T)	Prostate	Regulate male reproductive system, muscle mass, bone density	Fuels growth via androgen-receptor signaling	AR + Most cases	Anti-androgens, GnRH agonists	PSA testing, Reducing T therapy	65–75	(106–108)
Progesterone	Ovarian	Prepares uterus for pregnancy	Suppresses ovarian epithelial cell proliferation	PR + Varies	Progestin therapy	Regular pelvic exams, birth control	50–60	(71, 109, 110)
	Breast	Prepares uterus for pregnancy	In combination with estrogen, affects breast tissue development	PR + ∼65% of cases	Combination hormone therapy	Breast exams, reduce prolonged HRT	50–70	(97, 110, 111)
Prolactin	Breast	Regulates milk production	Excessive levels increase breast cell proliferation	PRLR + Varies	Dopamine agonists	Regular breast exams, reduce antipsychotic drugs	40–60	(112–115)
Luteinizing hormone (LH)	Testicular	Stimulates testosterone production	In some cancers, can stimulate cancer cell growth	LHR + Varies	LH-releasing hormone therapy	Monthly testicular exams	30–35	(116, 117)
Follicle-stimulating hormone (FSH)	Ovarian	Regulates egg maturation	Involved in early stages of follicle development; potential role in tumorigenesis	FSHR + Varies	Surgical removal, hormone therapies	Annual gynecological exams	40–50	(118–120)

The presented table provides a comprehensive overview of select sex hormones and their associations with specific cancer types. For each hormone, the table lists the cancers with which it's most commonly associated, its primary role in standard physiological processes, its prevalence across genders (male vs. female), the general mechanism by which the hormone may influence cancer development or progression, potential therapeutic interventions targeting the hormonal pathway, potential preventative measures, and the common age range of onset for each associated cancer. the term “Varies” within columns denotes that the presence of these receptors can differ widely among individual cases, cancer subtypes, or across populations.

### 3.1 Sex hormone levels and treatment efficacy

Hormonal status can be a major determinant in how patients respond to cancer treatment. This is observed in hormone-sensitive cancers like breast and prostate, but also others, such as melanoma (121) and colon cancer (122, 123). Breast cancer's deep-rooted connection to hormones serves as a clear illustration. Therapies that target estrogen pathways, such as Tamoxifen or aromatase inhibitors, are beneficial for patients with ER+ tumors. However, their efficacy diminishes in those with lower estrogen levels (124). Additionally, combination strategies like radiation therapy and Fulvestrant have shown improved immunotherapy responses in ER+ breast cancer (27). In prostate cancer, the success of androgen deprivation therapy (ADT) is built on the understanding that these tumors often rely on androgens (125). However, outcomes with ADT can vary significantly depending on baseline testosterone levels. Additionally, long-term ADT usage has been linked with an increased risk of colorectal cancer (16, 126). As personalized medicine becomes a standard, understanding how sex hormones influence treatment efficacy is paramount (127). Additionally, advancements in biomedical imaging, nanotechnology, COVID-19 research, and immunotherapy hold potential to enhance the efficacy of sex-specific cancer therapies. These fields, while not yet fully integrated into sex-specific treatment paradigms, offer promising avenues for exploring and understanding the impact of hormones on cancer. While their direct contribution to sex-specific therapy is still emerging, there is no doubt that these interdisciplinary domains will significantly influence future developments in this area (42–46, 128–164).

### 3.2 Sex hormone-targeting therapies

Sex hormones are undeniably influential in targeted cancer treatments. Over the years, strategies that modulate these hormones or their receptors have shown promise across various cancers. Tamoxifen, for instance, has become a cornerstone for ER+ breast cancer due to its ability to antagonize estrogen receptors (165), significantly reducing recurrence rates. Aromatase inhibitors, such as letrozole and anastrozole, by decreasing estrogen production, hinder tumor growth (166). In prostate cancer, anti-androgens like bicalutamide and enzalutamide have revolutionized treatment by interrupting vital growth signals (39, 167). Despite their efficacy, these therapies are not without challenges. Tamoxifen might elevate the risk of endometrial cancer in post-menopausal women, while aromatase inhibitors can lead to bone loss (31, 168). While anti-androgens are transformative, they come with cardiovascular risks and might eventually lead to resistance (25, 169). Ongoing studies, such as the development of selective estrogen receptor degraders (SERDs) that interact with ER-positive immune cells, show promise in enhancing the response to immune checkpoint inhibitors in breast cancer (24).

### 3.3 Disparities in treatment outcomes between males and females

Sex hormones intricately link to the differences observed in treatment outcomes between males and females (170). Although breast cancer is mainly found in women, its rare occurrence in men brings unique challenges (38). Similarly, prostate cancer’s androgen signaling complexities provide insights that can be compared with certain breast cancers in females. Furthermore, research in lung cancer has unveiled potential gender differences in treatment responses. Some studies suggest women may have more favorable outcomes with specific targeted therapies than men (171, 172). Additionally, hormone-targeting agents can induce gender-specific side effects; for instance, women on aromatase inhibitors might deal with symptoms of estrogen deficiency, whereas men on anti-androgen treatments might develop gynecomastia (173). Conclusively understanding the interplay among gender, sex hormones, and cancer treatments is vital for optimizing patient care.

## 4 Therapeutic implications

### 4.1 Hormone replacement therapy (HRT) and cancer outcomes

The therapeutic landscape for hormone-sensitive cancers is vast and continually evolving. HRT, which is used in the management of menopausal symptoms in women and conditions such as hypogonadism in men, remains a contentious subject in oncology (174). Although HRT has been seen to offer protective effects against conditions like osteoporosis and improve quality of life, its relationship with cancer is complex (175). Some studies, like the Women’s Health Initiative, have shown an increased risk of breast cancer with combined estrogen-progestin therapy, while estrogen-only therapy might have a protective effect. Additionally, other studies have highlighted an increased ovarian cancer risk with prolonged HRT (165, 176), whereas evidence suggests a protective effect against colorectal cancer (37). The effects of estrogen and its receptors on the immune tumor microenvironment in breast cancer (25) further complicate this dynamic. For breast cancer survivors, the use of HRT has been a concern, especially in potentially reactivating dormant cancer cells or progressing undetected micrometastases. When discussing testosterone replacement therapy (TRT) in men, concerns analogous to HRT in women arise, particularly regarding prostate cancer risk, which is further complicated with evidence suggesting that long-term androgen deprivation therapy for prostate cancer might increase colorectal cancer risk (16). In summary, the correlation between HRT and cancer remains intricate, highlighting the necessity for individualized treatment plans.

### 4.2 Personalized therapies based on hormonal levels

The trend in oncological treatments is shifting from a one-size-fits-all approach to personalized strategies, recognizing the importance of individual variations. For instance, the role of hormones in influencing anticancer immune surveillance elements underscores the importance of tailoring therapies based on hormonal profiles (38). Treatments for breast cancer, like the combination of radiation therapy and Fulvestrant, which has shown enhanced immunotherapy response in ER+ breast cancers (27), are reliant on the patient's hormonal environment. Similar strategies are evident in other cancers. For instance, estrogen signaling plays a role in myeloid cells promoting immune suppression in melanoma, suggesting that targeting these pathways might improve immunotherapy response (23). As we move forward, treatments might integrate hormonal profiling with genetic and molecular markers, paving the way for more personalized and effective therapies. Advanced biomedical imaging techniques have shed light on the nuanced interactions between sex hormones and tumor microenvironments, providing invaluable insights into differential therapy responses in male and female patients and paving the way for individualized treatment regimens (45, 141, 153, 154).

### 4.3 Future treatments targeting hormonal pathways

The recognition of the interplay between hormones and cancer progression has provided an avenue for new therapeutic breakthroughs. There is increasing evidence suggesting potential new treatments, such as the ERβ agonist LY500307 that suppresses lung cancer metastasis by activating antitumor neutrophils (58), or the utilization of newly designed SERDs that interact with ER-positive immune cells to improve the response to immune checkpoint inhibitors in breast cancer (24). The growing interest extends to targeting enzymes involved in hormone synthesis and metabolism. As biotechnological advancements like antibody-drug conjugates emerge, these allow for a more targeted delivery to hormone-sensitive tumors. The potential of gene therapy in modulating specific hormonal pathways also presents promising avenues for treatment, especially when considering how sex steroid hormones might interact with DNA repair components affecting genotoxic therapy response (165). Additionally, insights into sex differences in immunotherapy responses (33) highlight the potential of novel combinations that might be more tailored to the individual’s hormonal landscape. As our comprehension of the hormonal pathways in cancer deepens, it opens the door to more innovative treatments, promising better outcomes for patients. In the realm of personalized medicine, biomaterials stand out by creating hormone-responsive matrices and scaffolds for tissue engineering, enabling a more in-depth study of hormone-tumor interactions and contributing to the development of innovative therapeutic interventions tailored to specific hormonal environments (132, 150, 157).

## 5 Conclusion

Sex hormones, as important regulators of numerous physiological processes, wield profound influence on the landscape of cancer therapy outcomes. This review underscores the nuanced relationship between these hormones and various cancers, revealing the intricate web of interactions that determine therapeutic efficacy. From the direct implications of estrogen, androgens, and progesterone on tumors such as those of the breast, prostate, ovaries, and endometrium to the broader effects on cancer treatment outcomes, the role of sex hormones is indisputably significant. Clinically, it is paramount to consider hormonal levels as influential determinants in therapeutic decision-making. Their influence on treatment responsiveness, especially in hormone-sensitive cancers, emphasizes the need for a tailored approach in therapeutic strategies. Moreover, the disparities witnessed in treatment outcomes between males and females further accentuate the criticality of this consideration. Yet, while strides have been made in recognizing and acting upon these insights, much remains to be explored. Personalized therapies based on an individual's hormonal profile and future treatments targeting hormonal pathways are avenues that hold immense promise. We strongly advocate for intensified research efforts in this domain, aiming to refine therapeutic approaches and develop strategies that cater to the individual intricacies of each patient. By delving deeper into the realm of sex hormones and their connections with cancer, the medical community stands poised to usher in an era of enhanced therapeutic precision and efficacy.
